# Mechanism of succinate efflux upon reperfusion of the ischaemic heart

**DOI:** 10.1093/cvr/cvaa148

**Published:** 2020-08-07

**Authors:** Hiran A Prag, Anja V Gruszczyk, Margaret M Huang, Timothy E Beach, Timothy Young, Laura Tronci, Efterpi Nikitopoulou, John F Mulvey, Raimondo Ascione, Anna Hadjihambi, Michael J Shattock, Luc Pellerin, Kourosh Saeb-Parsy, Christian Frezza, Andrew M James, Thomas Krieg, Michael P Murphy, Dunja Aksentijević

**Affiliations:** 1 MRC Mitochondrial Biology Unit, University of Cambridge, The Keith Peters Building, Cambridge Biomedical Campus, Hills Road, Cambridge CB2 0XY, UK; 2 Department of Medicine, University of Cambridge, Addenbrookes Hospital, Hills Road, Cambridge CB2 0QQ, UK; 3 Department of Surgery, University of Cambridge, Cambridge NIHR Biomedical Research Centre, Biomedical Campus, Hills Road, Cambridge CB2 0QQ, UK; 4 MRC Cancer Unit, University of Cambridge, Hutchison/MRC Research Centre, Cambridge Biomedical Campus, PO Box 197, Cambridge CB2 0XZ, UK; 5 Bristol Medical School and Translational Biomedical Research Centre, Faculty of Health Science, University of Bristol, Level 7, Bristol Royal Infirmary, Upper Maudlin Street, Bristol BS2 8HW, UK; 6 Département de Physiologie, Université de Lausanne, 7 Rue du Bugnon, 1005 Lausanne, Switzerland; 7 King’s College London, British Heart Foundation Centre of Excellence, The Rayne Institute, St Thomas’ Hospital, Lambeth Palace Road, London SE1 7EH, UK; 8 Centre de Résonance Magnétique des Systèmes Biologiques, UMR5536 CNRS, LabEx TRAIL-IBIO, Université de Bordeaux, 146 Rue Leo Saignat, Bordeaux 33076, France; 9 Inserm U1082, Université de Poitiers, 2 Rue de la Miletrie, Poitiers 86021, France; 10 William Harvey Research Institute, Barts and The London School of Medicine and Dentistry, Queen Mary University of London, Charterhouse Square, London, UK; 11 Centre for inflammation and Therapeutic Innovation, Queen Mary University of London, Charterhouse Square, London, UK

**Keywords:** Ischaemia/reperfusion injury, Succinate, MCT1 transporter, Mitochondria, SUCNR1

## Abstract

**Aims:**

Succinate accumulates several-fold in the ischaemic heart and is then rapidly oxidized upon reperfusion, contributing to reactive oxygen species production by mitochondria. In addition, a significant amount of the accumulated succinate is released from the heart into the circulation at reperfusion, potentially activating the G-protein-coupled succinate receptor (SUCNR1). However, the factors that determine the proportion of succinate oxidation or release, and the mechanism of this release, are not known.

**Methods and results:**

To address these questions, we assessed the fate of accumulated succinate upon reperfusion of anoxic cardiomyocytes, and of the ischaemic heart both *ex vivo* and *in vivo*. The release of accumulated succinate was selective and was enhanced by acidification of the intracellular milieu. Furthermore, pharmacological inhibition, or haploinsufficiency of the monocarboxylate transporter 1 (MCT1) significantly decreased succinate efflux from the reperfused heart.

**Conclusion:**

Succinate release upon reperfusion of the ischaemic heart is mediated by MCT1 and is facilitated by the acidification of the myocardium during ischaemia. These findings will allow the signalling interaction between succinate released from reperfused ischaemic myocardium and SUCNR1 to be explored.

## 1. Introduction

**Figure 1:**
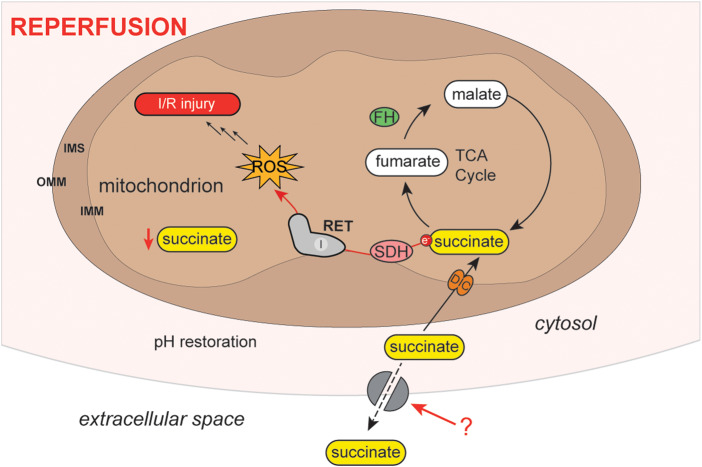
Schematic of metabolite changes occurring during reperfusion. Upon reperfusion, succinate is oxidized producing ROS fed by succinate re-entry into mitochondria. We favour RET at complex I as the mechanism of this ROS production. Some of the succinate is also released from the cell. DIC, dicarboxylate carrier; FH, fumarate hydratase; IMM, inner mitochondrial membrane; IMS, inter-membrane space; I/R, ischaemia/reperfusion; OMM, outer mitochondrial membrane; RET, reverse electron transport; ROS, reactive oxygen species; SDH, succinate dehydrogenase; TCA, tricarboxylic acid.

Succinate accumulates several-fold in a range of ischaemic tissues, including the heart.^1–5^ Upon reperfusion, the succinate levels very rapidly (<1.5–2 min) return to baseline values.^3^^,^^4^ A proportion of this accumulated succinate is oxidized by the mitochondrial respiratory chain, contributing to the formation of the reactive oxygen species (ROS), superoxide (*Figure 1*).^4^ This ROS production initiates a cascade of damage that culminates in ischaemia/reperfusion (I/R) injury.^4^ In addition to its oxidation, upon reperfusion a significant amount of the accumulated succinate is released from the heart into the circulation.^2^^,^^6^ The succinate accumulated during ischaemia is thought to move from the mitochondria to the cytosol, catalysed by the dicarboxylate carrier in exchange for malate.^7^ Therefore, upon reperfusion of the ischaemic heart, the cytosolic succinate has two fates—it either re-enters mitochondria as a respiratory substrate to drive ROS production or effluxes from the cell (*Figure 1*). Efflux of succinate from the ischaemic heart upon reperfusion has been demonstrated *ex vivo* in the mouse heart and in human hearts *in vivo* during primary percutaneous coronary intervention on ST-elevated myocardial infarction (STEMI) patients.^2^^,^^6^ However, the mechanism of succinate release is unknown.

Succinate accumulation is a conserved signature of ischaemia in different organs and species,^1–5^ suggesting that its release upon reperfusion may be a signal of tissue ischaemia and/or damage. Furthermore, there is a G-protein-coupled succinate receptor (SUCNR1) that can respond to the succinate released into the circulation (*Figure 7*).^8–10^ SUCNR1 is highly expressed on the surface of immune cells and its ligation has been associated with a range of both pro- and anti-inflammatory phenotypes, depending on context.^8^^,^^11–13^ In addition, succinate release into the circulation may have other effects in addition to signalling from ischaemic tissue, for example, succinate was shown to activate thermogenesis by brown adipose tissue.^14^ Together these findings suggest that succinate released from ischaemic tissues into the circulation may promote a range of responses, such as the infiltration of immune cells and thereby contribute to the pathology and/or resolution of I/R injury (*Figure 7*).^6^^,^^9^^,^^15^

To address the mechanism of succinate release into the circulation during reperfusion, we assessed the efflux of succinate from ischaemic cardiomyocytes, mouse hearts *ex vivo* and *in vivo*, and in a pig model of myocardial infarction (MI). We show that succinate was one of only a few metabolites released upon reperfusion of the ischaemic heart. Furthermore, succinate efflux was mediated by the monocarboxylate transporter 1 (MCT1) and acidification of the myocardium during ischaemia enhanced release upon reperfusion. This understanding of how succinate is released upon reperfusion of ischaemic organs has translational implications for targeting succinate signalling following MI.

## 2. Methods

All experiments were performed under UK Home Office Licences and conducted according to the Animals Scientific Procedures Act 1986 (UK) and directive 2010/63/EU of the European Parliament guidelines on the protection of animals used for scientific purposes. All experiments were approved by the Institutional Animal Welfare and Ethical Review Body. C57BL/6J male mice (∼25 g, 8–12 weeks old, *n* = 102) were from Charles River, UK. The *MCT1*^+/−^ mice (8–12 weeks old) were initially generated by homologous recombination^16^ and bred to produce MCT1^+/−^ and corresponding MCT1^+/+^ littermate controls. Female Wistar rats (∼250 g, 10–12 weeks old) were from Charles River, UK. All mice and rats were kept in individually ventilated cages with a 12 h light–dark cycle, controlled humidity and temperature (20–22°C), fed standard chow and water *ad libitum*. Experiments in pig were carried out under Home Office Project Licence No 7008975 at the University of Bristol Translational Biomedical Research Centre (TBRC), Bristol, UK, an advanced facility for large animal research (http://www.bristol.ac.uk/health-sciences/research/tbrc/).

For Langendorff perfusions, mice were administered terminal anaesthesia via intra-peritoneal pentobarbitone injection (∼140 mg/kg body weight). For *in situ* I/R, mice were anaesthetized with isoflurane (2 × minimum alveolar concentration and O_2_ at 2 L/min Abbott Laboratories, USA) before performing a laparotomy and administering 100 µL heparin bolus (100 iU; Leo Pharma A/S, Denmark). Mice were culled via exsanguination by division of the abdominal inferior vena cava (IVC) and aorta. For the acute murine MI model, mice were anaesthetized throughout the procedure with sodium pentobarbital (70 mg/kg body weight) and culled via exsanguination by division of the abdominal IVC. For isolation of adult cardiomyocytes, mice were culled by cervical dislocation (no anaesthetic used). For isolation of rat heart mitochondria (RHM), rats were culled by cervical dislocation (no anaesthetic used). In porcine MI model, landrace female pigs were premedicated with intramuscular injection of ketamine (10 mg/kg) and dexmedetomidine (15 µg/kg); for general anaesthesia, IV boluses of propofol (1 mg/kg) were used followed by isoflurane in oxygen with the vaporizer set at 2% for maintenance. At the end of the experiment, pigs were terminated by administration of 2 L cold cardioplegia solution via the aorta at a delivery pressure of 300 mmHg.

### 2.1 Animal I/R experimental models

#### 2.1.1 Langendorff-perfused mouse hearts

Mice were administered terminal anaesthesia via intra-peritoneal pentobarbitone injection (∼140 mg/kg body weight). While anaesthetics such as pentobarbitone can affect mitochondrial function, in our experiments the effects of inhibitors and other interventions are compared with controls using identical anaesthetic regimes. Beating hearts were rapidly excised, cannulated, and perfused in isovolumic Langendorff mode at 80 mmHg pressure maintained by an St. Thomas Hospital (STH) peristaltic pump controller feedback system (AD Instruments, UK), with phosphate-free Krebs–Henseleit (KH) buffer continuously gassed with 95% O_2_/5% CO_2_ (pH 7.4, 37°C) containing (in mM) NaCl (116), KCl (4.7), MgSO_4_.7H_2_O (1.2), NaHCO_3_ (25), CaCl_2_ (1.4), and glucose (11). Cardiac function was assessed using a fluid-filled cling-film balloon inserted into the left ventricle (LV) connected via a line to a pressure transducer and a Powerlab system (AD Instruments). The volume of the intraventricular balloon was adjusted using a 1.0 mL syringe to achieve an initial LV diastolic pressure (LVDP) of 4–9 mmHg. Functional parameters [systolic pressure (SP), end-diastolic pressure (DP), heart rate, coronary flow, perfusion pressure] were recorded using LabChart software v.7 (AD Instruments) throughout the experiment. LVDP was calculated from the difference between SP and DP. After 20 min equilibration, hearts were subjected to 20 min global ischaemia prior to reperfusion. Perfusate was collected in 1 min intervals for the first 6 min of reperfusion and snap frozen in liquid nitrogen. Where inhibitors were used, these were added in the reperfusion buffer, with the heart reperfused for 6 min containing the inhibitors throughout (unless specified otherwise). Hearts were immediately clamp frozen using Wollenberger tongs pre-cooled in liquid nitrogen either after equilibration, ischaemia, or the reperfusion period (6 min) and stored at –80°C until further analysis.

#### 2.1.2 *In situ* mouse heart I/R

Mice were anaesthetized with isoflurane (2 × minimum alveolar concentration and O_2_ at 2 L/min; Abbott Laboratories) before performing a laparotomy and administering 100 µL heparin bolus (100 iU; Leo Pharma A/S). Mice were exsanguinated by division of the abdominal IVC and aorta.

Global ischaemia was maintained within the body for 20 min, with physiological temperature (37°C) maintained using a core-temperature controlled heating-mat. During ischaemia, fine borosilicate tubing was inserted into the root of the aorta and a collection tube inserted into the IVC lumen. Immediately before reperfusion, the superior vena cava, pulmonary artery, and haemiazygos vein were clamped to prevent back flow from the right atrium on flush. The heart was retrograde perfused with phosphate-free KH buffer (37°C) at a flow rate of 1 mL/min. Perfusate was collected at 1 min intervals for 6 min and the heart clamp frozen.

#### 2.1.3 Porcine MI model

Landrace female pigs (*n* = 3; 5–6 months, median weight 62.5 kg) were premedicated with intramuscular injection of ketamine (10 mg/kg) and dexmedetomidine (15 µg/kg); for general anaesthesia, IV boluses of propofol (1 mg/kg) were used followed by isoflurane in oxygen with the vaporizer set at 2% for maintenance. Mechanical ventilation targets of tidal volume of 10–20 mL/kg and a respiratory rate of 10–20 breaths/min were used, aiming for end-tidal carbon dioxide between 35 and 45 mmHg. Full monitoring included electrocardiogram, invasive arterial blood pressure, temperature, saturation, and central venous pressure via a line in the central jugular vein (JV). Following median sternotomy and heparinization (150 IU/kg), a 5–0 prolene suture was passed around the proximal left anterior descending (LAD) artery just distal to the first diagonal branch; a catheter was inserted into the coronary sinus (CS) under direct vision and a sampling line was inserted into the aortic root (AR). Under stable conditions the LAD was gently snared to start a period of 60 min of ischaemia, after which the snare was released to allow reperfusion for another period of 60 min before termination and myocardial sampling. Blood was drawn serially from JV, CS, and AR at the following time points: baseline, before intervention, and at 5, 15, 30, and 60 min during ischaemia and at 1, 3, 5, 15, 30, and 60 min of reperfusion.

#### 2.1.4 *In vivo* murine MI model

The LAD coronary artery was occluded to induce MI in an acute open chest, *in situ* mouse model as described previously^17^ to assess the effects of AR-C141990 on I/R injury. Briefly, mice were anaesthetized by administration of sodium pentobarbital (70 mg/kg intraperitoneally), endotracheally intubated, ventilated with 3 cm H_2_O positive end-expiratory pressure and kept at 37°C using a rectal thermometer-controlled heatpad (TCAT-2LV, Physitemp, USA).

Ventilation frequency was maintained at 110 breaths/min, with tidal volume between 125 and 150 µL. The heart was exposed and a suture was placed around the prominent branch of the LAD and passed through a small plastic tube used to initiate ischaemia by pressing the tube against the heart surface to occlude the LAD. Mice were subjected to 30 min of ischaemia and 120 min of reperfusion, after reperfusion, hearts were stained with Evans Blue and 2% triphenyltetrazolium chloride and blindly analysed by an independent researcher.

### 2.2 Cardiomyocyte I/R model

#### 2.2.1 Isolation of adult primary mouse cardiomyocytes

Adult primary mouse cardiomyocytes were isolated as described previously.^18^ Mice were culled by cervical dislocation before rapidly excising the heart and cannulating via the aorta in ice-cold, sterile perfusion buffer [in mM: NaCl (113), KCl (4.7), KH_2_PO_4_ (0.6), Na_2_HPO_4_ (0.6), MgSO_4_.7H_2_O (1.2), NaHCO_3_ (12), KHCO_3_ (10), HEPES sodium salt (0.922), taurine (30), 2,3-butanedione monoxime (10), and glucose (5.5)]. Hearts were retrogradely perfused for 5 min with perfusion buffer (37°C) to clear residual blood, then the hearts were digested by perfusing digestion buffer [30 mL perfusion buffer supplemented with 5 mg Liberase (Roche, UK) and 12.5 µM CaCl_2_] for 20 min. After digestion, the heart was removed from the cannula and carefully broken apart with tweezers and gentle pipetting in 4 mL digestion buffer. The cell suspension was transferred to a 15 mL centrifuge tube before making up to 10 mL with stop buffer (37°C) [10% (v/v) foetal bovine serum in perfusion buffer] and allowed to pellet by gravity for 10 min at RT. After pelleting, the supernatant was removed and the cells were resuspended in sequentially increasing Ca^2+^ concentrations (62 µM, then 212 µM, then 1 mM) in 5 mL stop buffer. Cells were subsequently resuspended in M199 media (Gibco, Thermo Fisher Scientific) supplemented with 2 mM L-carnitine, 5 mM creatine, 5 mM taurine, 25 µM blebbistatin, 100 IU/mL penicillin, and 100 IU/mL streptomycin and plated (1 × 10^5^ cells/dish) in laminin-coated (0.1 mg/mL from Engelbreth–Holm–Swarm murine sarcoma basement membrane; Sigma Aldrich, UK) glass dishes.

#### 2.2.2 Anoxic cardiomyocyte incubations

Anoxic incubations were carried out using an anaerobic chamber (0.4 ppm O_2_; Belle Technologies, UK). Equipment and solutions were degassed overnight in the transfer compartment of the anoxic chamber before the experiment was performed.

Experiments were performed in Tyrode’s buffer [in mM: NaCl (137), KCl (5.4), MgCl_2_ (0.4), HEPES (10), glucose (10), CaCl_2_ (1), pH 7.4]. The MCT1 inhibitor (MCTi) AR-C141990 (Tocris, Biotechne) was used at a concentration of 10 µM in Tyrode’s buffer. Cardiomyocytes were plated (1 × 10^5^ cells/plate), washed once with Tyrode’s buffer (37°C) before 2 mL fresh Tyrode’s buffer were added to each dish. Anoxia was induced for different time points by placing dishes in the anaerobic chamber on a 37°C heat block, before the cells were lysed under anoxia, transferred to Eppendorf tubes and snap frozen on dry ice. Cells were reperfused by removing from the anaerobic chamber, replacing buffer with fresh Tyrode’s buffer and incubating (37°C, 15 min) before lysing cells for subsequent succinate analysis.

### 2.3 Metabolite measurement by mass spectrometry

#### 2.3.1 Succinate extraction

For tissue samples, frozen tissues were weighed out on dry ice to achieve ∼25 mg of each tissue sample. Tissues were extracted with 25 µL/mg MS extraction buffer [50% (v/v) methanol, 30% (v/v) acetonitrile, and 20% (v/v) H_2_O], supplemented with 1 nmol of [^13^C_4_]-succinate (Sigma Aldrich) in a pre-chilled Precellys tube (hard tissue homogenizing CK28-R—2 mL; Bertin Instruments, France). Tissues were homogenized using a Precellys 24 tissue homogenizer (6500 rpm, 15 s; Bertin Instruments) and then immediately placed back on dry ice for 5 min. Samples were re-homogenized (6500 rpm, 15 s) and again placed on dry ice to cool before agitating in a shaking heat block (1400 rpm, 15 min, 4°C; Thermo Fisher Scientific, UK) in a cold room (4°C) and then incubating (−20°C, 1 h). Samples were subsequently centrifuged (17 000 × *g*, 10 min, 4°C) and the supernatant transferred to a pre-chilled microcentrifuge tube and recentrifuged (17 000 × *g*, 10 min, 4°C). The resulting supernatants were transferred to pre-cooled MS vials which were stored at −80°C until analysis for succinate by liquid chromatography–tandem mass spectrometry (LC–MS/MS).

For perfusate from the Langendorff heart and plasma samples, the perfusate or plasma was centrifuged (17 000 × *g*, 10 min, 4°C) before extracting 50 µL in 750 µL MS extraction buffer supplemented with 1 nmol [^13^C_4_]-succinate. The samples were agitated in the cold (1400 rpm, 15 min, 4°C; Thermo Fisher Scientific) before incubating (−20°C, 1 h). The samples were centrifuged (17 000 × *g*, 10 min, 4°C), then the supernatant transferred to a new tube and recentrifuged. The clear supernatant was transferred to MS vials and stored at −80°C until analysis.

For adult primary cardiomyocytes, cardiomyocytes were extracted in 500 µL MS extraction buffer supplemented with 1 nmol [^13^C_4_]-succinate and agitated in the cold (1400 rpm, 15 min, 4°C; Thermo Fisher Scientific) before incubating (−20°C, 1 h). The samples were centrifuged (17 000 × *g*, 10 min, 4°C), then the supernatant transferred to a new tube and recentrifuged. The clear supernatant was transferred to MS vials and stored at −80°C until analysis.

#### 2.3.2 Quantification of succinate

LC–MS/MS analysis of succinate was performed using an LCMS-8060 mass spectrometer (Shimadzu, UK) with a Nexera X2 UHPLC system (Shimadzu). Samples were stored in a refrigerated autosampler (4°C) before injection of 5 μL using a 15 μL flowthrough needle. Separation was achieved using a SeQuant^®^ ZIC^®^-HILIC column (3.5 μm, 100 Å, 150 mm × 2.1 mm, 30°C column temperature; MerckMillipore, UK) with a ZIC^®^-HILIC guard column (200 Å, 1 mm × 5 mm). A flow rate of 200 μL/min was used with mobile phases of buffer A: 10 mM ammonium bicarbonate and buffer B: 100% acetonitrile. A gradient of 0–0.1 min, 80% MS buffer B; 0.1–4 min, 80–20% B; 4–10 min, 20% B, 10–11 min, 20–80% B; 11–15 min, 80% B was used. The mass spectrometer was operated in negative ion mode with multiple reaction monitoring and spectra were acquired using LabSolutions software (Shimadzu), with compound quantities calculated from relevant standard curves in MS extraction buffer and comparing against [^13^C_4_]-succinate internal standard.

#### 2.3.3 Metabolomic analysis by LC–MS

Samples were extracted as for succinate quantification but ^13^C-succinate omitted and 5 µM *d_8_*-valine added instead. LC–MS analyses were performed on a Q Exactive Orbitrap (Thermo Scientific) mass spectrometer coupled to an Ultimate 3000 UHPLC system (Dionex). The LC system was fitted with either a ZIC-HILIC column (150 mm × 4.6 mm) or a ZIC-pHILIC column (150 mm × 2.1 mm) and respective guard columns (20 mm × 2.1 mm) (all Merck, Germany). The metabolites were eluted with previously described gradients.^19^ The mass spectrometer was operated in full MS and polarity switching mode. Samples were randomized in order to avoid bias due to machine drift and processed blindly. The acquired spectra were analysed using XCalibur Qual and XCalibur Quan Browser software (Thermo Fisher Scientific) by referencing to an internal library of compounds.

### 2.4 Isolation of rat heart mitochondria 

Rat heart mitochondria (RHM) were isolated as described previously.^20^ Briefly, freshly excised rat hearts were homogenized in STEB buffer [250 mM sucrose, 5 mM Tris, 1 mM EGTA, 0.1% (w/v) bovine serum albumin (BSA), pH 7.4] using a Dounce homogenizer. Homogenates were centrifuged (3000 × *g*, 5 min, 4°C) to pellet nuclei and unbroken cells. The resulting supernatant was then centrifuged (10 000 × *g*, 10 min, 4°C) to pellet mitochondria. Pelleted mitochondria were resuspended in STEB buffer and recentrifuged (10 000 × *g*, 10 min, 4°C). The pelleted mitochondria were resuspended in STEB buffer with the BSA omitted (400 µL/heart) and the mitochondrial protein quantified by bicinchoninic (BCA) assay (Thermo Fisher Scientific).

### 2.5 Measurement of ROS production by RET

ROS production by reverse electron transport (RET) was measured by following the conversion of Amplex Red to resorufin.^20^ Isolated RHM were incubated in KCl buffer (120 mM KCl, 10 mM HEPES, 1 mM EGTA, pH 7.4; 37°C) supplemented with Amplex Red (12.5 µM; Invitrogen, Thermo Fisher Scientific), horseradish peroxidase (20 µg/mL), BSA (200 µg/mL), superoxide dismutase (40 µg/mL), and succinate (0–10 mM) in a 96-well plate. Resorufin fluorescence was detected by λ_ex_= 570 nm and λ_em_= 585 nm and calibrated against known concentrations of hydrogen peroxide (46.6 M^−1^cm^−1^ at 240 nm).

### 2.6 Statistics and experimental design

All data in figures are presented as mean ± standard error of the mean (SEM), unless stated otherwise in the figure legend. Statistical analysis was performed using either one- or two-way ANOVA with the suitable *post hoc* correction for multiple comparisons described in the figure legend. Where only two groups were compared, statistical significance was assessed by two-tailed Student’s unpaired *t*-test. A *P*-value of <0.05 was considered significant. Statistics were calculated in Prism 8.0 software (GraphPad Software Inc., USA).

## 3. Results

### 3.1 Succinate efflux upon reperfusion of an *ex vivo* ischaemic heart

**Figure 2:**
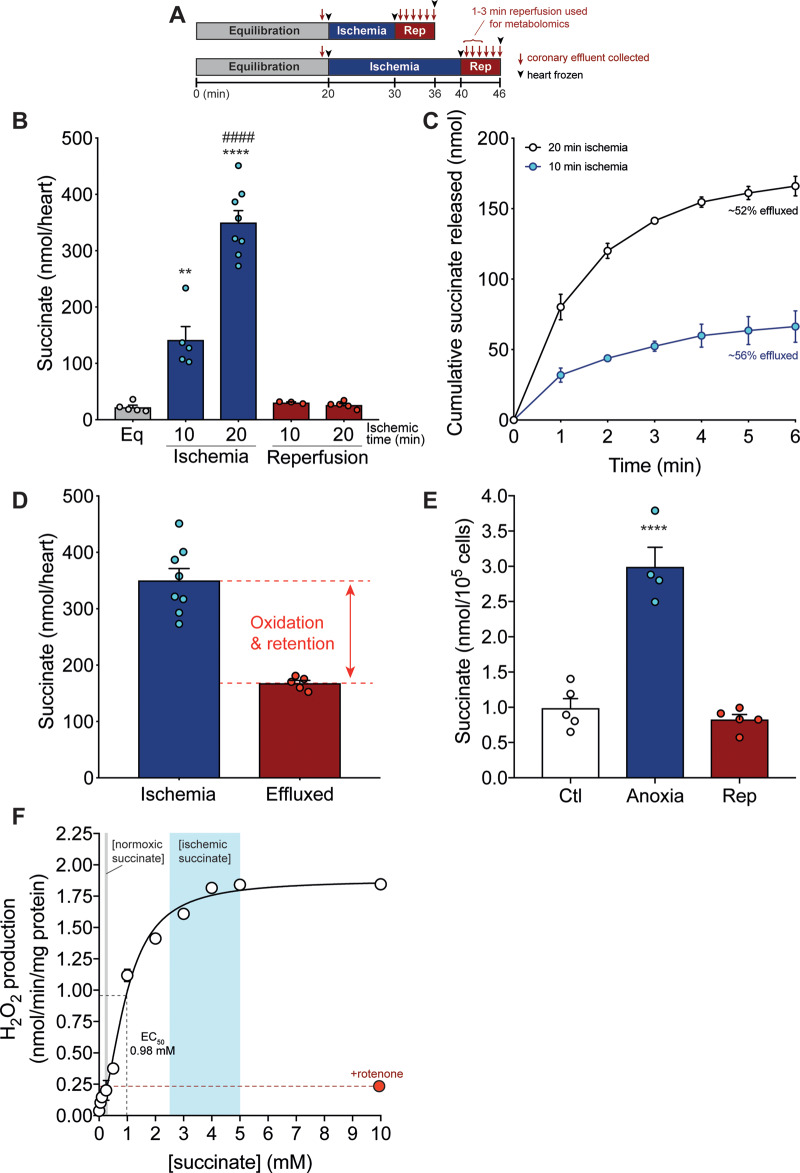
(*A–F*) Succinate accumulation and efflux from the ischaemic Langendorff heart and primary cardiomyocytes. (*A*) Langendorff model experimental design of ischaemia and reperfusion. (*B*) Time-dependent succinate accumulation in a Langendorff-perfused mouse heart exposed to 10 or 20 min global no-flow ischaemia ± 6 min reperfusion (mean ± SEM, *n* = 3–8). Statistical significance was assessed by one-way ANOVA with Tukey’s *post hoc* test (**P <* *0.01, ****P <* *0.0001 *relative to equilibration, ^####^*P *<* *0.0001 ^#^relative to 10 min ischaemia). (*C* and *D*) Succinate efflux from the Langendorff heart exposed to 10 or 20 min ischaemia over the first 6 min of reperfusion (*C*) and compared to the succinate levels achieved in the heart exposed to 20 min ischaemia (*D*) (mean ± SEM, *n* = 5–8). (*E*) Succinate accumulation and efflux in primary cardiomyocytes exposed to anoxia (1 h) ± reperfusion (15 min) (mean ± SEM, *n* = 4–6). Statistical significance was assessed by one-way ANOVA with Dunnett’s *post hoc* test ****P <* *0.0001, relative to control (Ctl) values. (*F*) ROS production by RET in isolated heart mitochondria. Isolated RHM were incubated with varying succinate concentrations and where indicated rotenone (0.5 µM), and the production of H_2_O_2_ measured by the conversion of Amplex Red to resorufin (mean ± SEM, *n* = 3). The EC_50_ for the dependence of ROS production by RET on succinate concentration is shown.

To quantify succinate efflux from the ischaemic heart upon reperfusion, we used the Langendorff isolated heart perfusion model. Mouse hearts following 20 min functional equilibration (LVDP 94 ± 3 mmHg, heart rate 453 ± 20 b.p.m., coronary flow 3.5 ± 0.14 ml/min mean ± SEM, *n* = 17) were then exposed to 20 min global, no-flow ischaemia, followed by reperfusion with oxygenated perfusion buffer (*Figure 2A*). Langendorff hearts exposed to ischaemia led to a 14-fold increase in succinate levels (*Figure 2B*), comparable to the succinate accumulation seen previously in the ischaemic heart *in vivo.*^4^^,^^5^ Upon subsequent reperfusion, tissue succinate levels returned to baseline (*Figure 2B)*. Halving the ischaemic time decreased succinate accumulation, but following reperfusion the levels of succinate were the same (*Figure 2B*). To see if any of the succinate that had accumulated during ischaemia was released from the heart upon reperfusion, we next measured succinate in the coronary effluent immediately after reperfusion (*Figure 2A*). Succinate was released from the myocardium into the circulation over the first 2 min of reperfusion, with little further release after 3 min (*Figure 2C*). Note that the quantification of succinate release is normalized to perfusate volume and is thus independent of the flow rate. Comparing the total amount of succinate in the perfusate to that in the heart at the onset of ischaemia, and correcting for baseline levels, showed that about half of the succinate accumulated during ischaemia was released (*Figure 2C and D*), as was shown previously by Brookes and colleagues.^6^ To confirm that this succinate efflux was largely from the cardiomyocytes, we used a murine primary adult cardiomyocyte model exposed to anoxia and reperfusion. Succinate accumulated significantly in anoxic cardiomyocytes and returned to baseline levels upon reperfusion (*Figure 2E*). After reperfusion, 1.3 ± 0.25 nmol succinate/10^5^ cells (mean ± SEM, *n* = 6) was released into the incubation medium. This succinate release is ∼60% of that accumulated within the cardiomyocytes during ischaemia (corrected for succinate remaining after reperfusion; *Figure 2E*) and is consistent with the amount released from the heart upon reperfusion (*Figure 2C and D*).

### 3.2 Dependence of ROS production by RET on succinate concentration

In our *ex vivo* system we found that after 20 min global ischaemia, the level of succinate per heart increased to ∼360–400 nmol succinate/heart, while in a previous study we found that 30 min global ischaemia of the heart *in situ* leads to ∼800 nmol succinate/heart.^5^ As the mouse hearts used in these studies weigh ∼184 ± 16 mg (*n* = 8, mean ± SEM), this corresponds to 2–4 µmol succinate/g wet weight. The water content of the rodent heart is 615 µL/g wet weight intracellular and 174 µL/g wet weight extracellular.^21^ Assuming that the succinate stays within the cells during ischaemia and is distributed roughly equally throughout the cell, this corresponds to an intracellular succinate concentration of 3–6 mM at the onset of reperfusion. Even if during ischaemia the succinate was also equally distributed between the intracellular and extracellular water, this would still give an intracellular succinate concentration of 2.5–5 mM at the onset of reperfusion. The level of succinate in the normoxic mouse hearts here is 22 ± 3 nmol/heart (mean ± SEM *n* = 5) and previously we reported a succinate level in the normoxic mouse heart of 34 nmol/heart.^5^ This corresponds to normoxic succinate levels of 120–185 nmol/g wet weight or an intracellular succinate concentration of 195–300 µM. Thus, even if we assume that the efflux of ∼50% of the intracellular succinate occurred immediately upon reperfusion, this would still leave mitochondria exposed to 1.5–3 mM succinate at the onset of reperfusion, decreasing down to ∼200–300 µM after 2–3 min. To see if these levels of succinate were sufficient to drive RET at complex I, we determined the dependence of complex I ROS production by RET in isolated heart mitochondria on succinate concentration (*Figure 2F*). This showed that RET production driven by succinate saturated at ∼4 mM with a half-maximal effective concentration (EC_50_) of ∼1 mM (*Figure 2F*). This ROS production was largely inhibited by the complex I inhibitor rotenone, which blocks RET but does not affect ROS production at complex III. This is consistent with the protective effect of rotenone on IR injury/infarct size and suggests that the ROS production measured here is essentially all generated by RET at complex I. Thus, the level of succinate present within the ischaemic heart at the onset of reperfusion is more than adequate to generate ROS by RET and this level of succinate must decrease by ∼80% before substantially affecting RET. Thus, the efflux of succinate from the tissue upon reperfusion does not impact on its ability to drive RET and cause pathological I/R injury.

### 3.3 Selectivity of succinate release upon reperfusion

**Figure 3:**
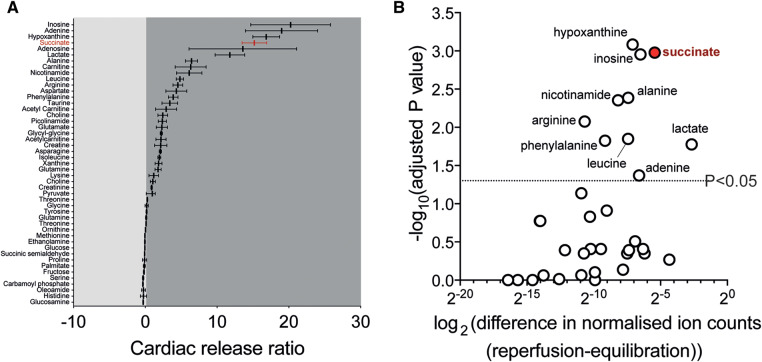
(*A* and *B*) Succinate is selectively effluxed from the ischaemic heart upon reperfusion. (*A*) Release of metabolites in the reperfusion coronary effluent (1–3 min) from the Langendorff heart, compared to equilibration coronary effluent expressed as cardiac release ratio [(reperfusion − equilibration)/equilibration]. (*B*) Plot showing −log_10_ of the adjusted *P-*value plotted against the difference in normalized ion counts between equilibration and 1–3 min reperfusion coronary effluent (*n* = 5). Plot generated in Prism 8.0 using multiple *t*-tests corrected for multiple comparisons using the Holm–Sidak method.

To determine whether the succinate release from the heart was selective, or part of a general metabolite release due to tissue damage upon reperfusion, the metabolome of the perfusate from the Langendorff hearts was assessed (*Figure 3A*). For this, we could quantify the levels of 47 metabolites in the perfusate from the normoxic heart under equilibration, as well as in the perfusate during the first 3 min reperfusion of the ischaemic heart (*Figure 2A*). We calculated the cardiac release ratio,^2^ the difference in metabolite levels between reperfusion and normoxia, normalized to the levels in the normoxic effluent (*Figure 3A*). This showed that only a few of the 47 metabolites measured were released upon reperfusion. To assess this further, we plotted the differences in metabolites in the perfusate between reperfusion and normoxia (*Figure 3B*). This demonstrated that only 10 metabolites showed a statistically significant release upon reperfusion. Among these were nicotinamide and the adenine nucleotide breakdown products hypoxanthine, adenine, and inosine which are known to accumulate during ischaemia.^4^^,^^5^^,^^22^ These metabolites are all neutral so they may diffuse passively through the cell membrane.^23^ In contrast, the other metabolites released—succinate, lactate, and some amino acids—are charged and will require transporters to leave the cell upon reperfusion. Among charged substrates the cardiac release ratio was highest for succinate (*Figure 3A*), indicating that succinate is a major charged metabolite released upon reperfusion of the ischaemic tissue probably via a selective transport pathway.

**Figure 4:**
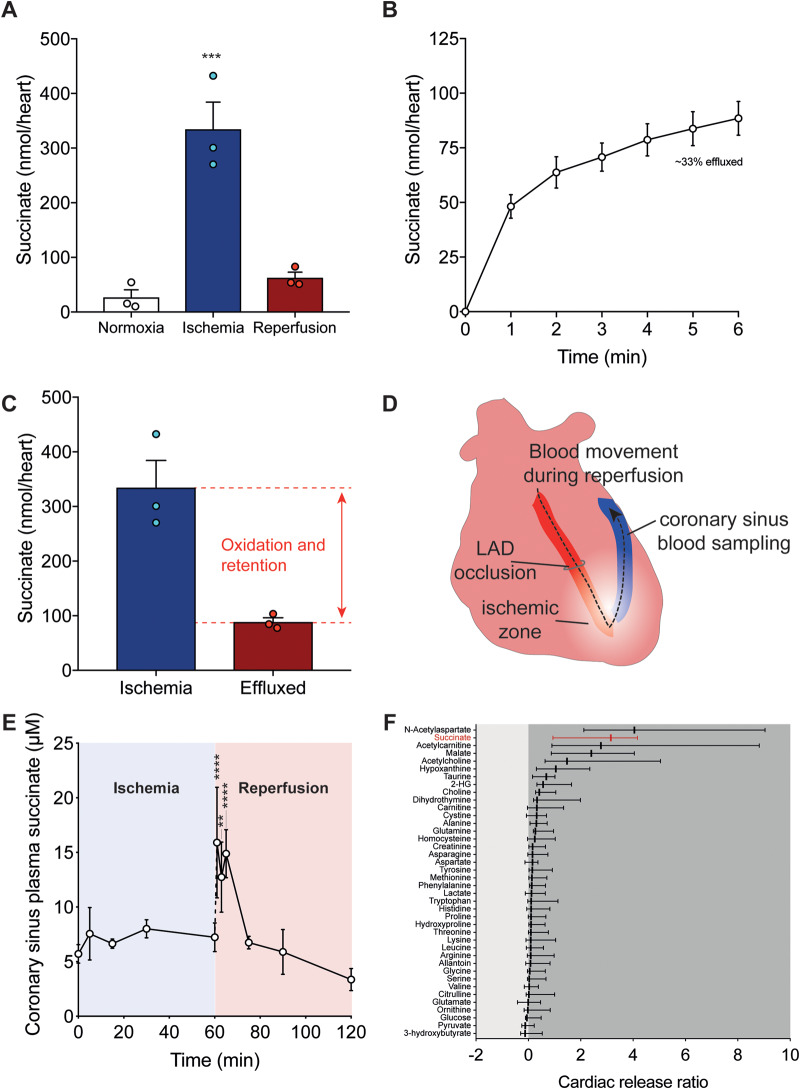
(*A–F*) Succinate accumulation and efflux from the ischaemic heart *in situ* and *in vivo*. (*A*) Succinate accumulates during 20 min ischaemia and rapidly returns to baseline values after 6 min reperfusion in a murine *in situ* perfusion model (mean ± SEM, *n* = 3). Statistical significance was assessed by one-way ANOVA with Dunnett’s *post hoc* test [***P <* *0.001, relative to control (Normoxia) values]. (*B* and *C*) Succinate efflux from the *in situ* perfused heart exposed to 20 min ischaemia over the first 6 min of reperfusion (*B*) and compared to the succinate levels achieved in the heart exposed to 20 min ischaemia (*C*) (mean ± SEM, *n* = 3). (*D*) Schematic of porcine MI model and CS blood sampling (*E*). Succinate is elevated during early reperfusion in the CS in a porcine MI model. The LAD was occluded by gentle snaring for 60 min before snare released and blood sampled (mean ± SEM, *n* = 3). Statistical significance was assessed by two-way ANOVA with Tukey’s *post hoc* test (**P <* *0.01, ****P <* *0.0001). (*F*) Release of metabolites in the CS at reperfusion (1–5 min) compared to AR blood expressed as cardiac release ratio [(CS − AR)/AR] in porcine MI model. LAD, left anterior descending.

As the Langendorff heart is perfused with an oxygenated, hyperglycaemic crystalloid buffer, we were uncertain how this might affect succinate accumulation, efflux, and consumption upon reperfusion compared to the *in vivo* situation. To address this potential concern, we assessed mouse hearts that were rendered ischaemic *in situ*, prior to which they had been supplied with normal blood. To do this we induced global no-flow ischaemia by exsanguination and then left the heart in the body for 20 min maintained at 37°C. Then, the blood vessels were clamped and the heart was flushed with Krebs buffer and the perfusate collected. During global *in situ* ischaemia, we found significant accumulation of succinate (*Figure 4A*) and flushing the heart with oxygenated buffer led to a return to baseline levels of tissue succinate (*Figure 4A*). Measurement of succinate in the perfusate following flushing showed loss of succinate from the heart to the perfusate over the first 2–3 min reperfusion (*Figure 4B*), very similar to that for the Langendorff hearts. Comparing the total amount of succinate released with that present at the onset of ischaemia and correcting for baseline levels showed that ∼33% of the succinate that was present in the tissue at the end of ischaemia was released upon flushing (*Figure 4C*). Thus, the succinate efflux seen in the *ex vivo* perfused heart is replicated by the more physiological *in situ* mouse heart.

To extend this analysis to a large animal model closer in size to human, we used a pig MI model of I/R in which a 5–0 prolene suture was passed around the coronary artery and gently snared to block blood flow and to hold the heart ischaemic for 60 min and then released to reperfuse the ischaemic tissue with oxygenated blood (*Figure 4D*). We assessed the efflux of succinate from the ischaemic tissue into the CS, which reflects any metabolites released by the ischaemic myocardium upon reperfusion, as well as that in the AR and in the JV (*Figure 4E* and Supplementary material online, *Figure S1*). Upon reperfusion, there was a large increase in the succinate levels in the CS during the first 5 min reperfusion (*Figure 4E*), but not in the blood from the AR or JV (Supplementary material online, *Figure S1*). To understand whether the levels of succinate released from the pig heart during reperfusion were significant compared to other metabolites, we measured the levels of 40 metabolites in the CS upon reperfusion and in the AR, enabling us to generate a cardiac release ratio (*Figure 4F*). This showed that only a few metabolites were released upon reperfusion of the ischaemic heart tissue of pigs undergoing MI and that prominent among them was succinate (*Figure 4F*).

### 3.4 Succinate efflux can be inhibited

**Figure 5:**
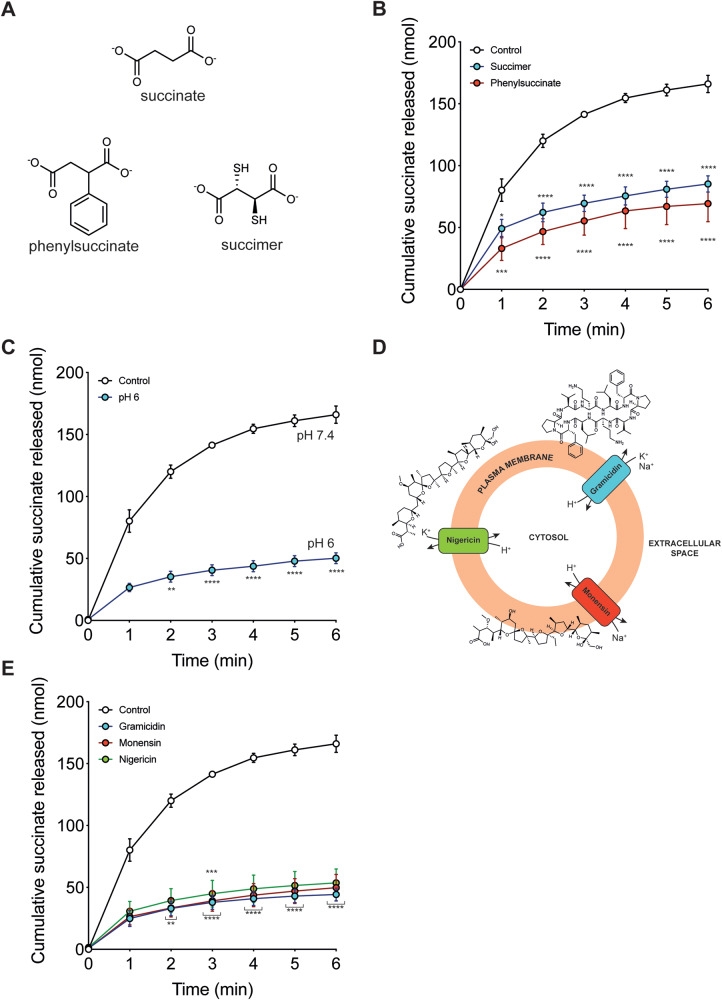
Modulating succinate efflux during reperfusion. (*A*) Succinate is structurally similar to phenylsuccinate and succimer. (*B* and *C*) Langendorff mouse hearts were exposed to 20 min global no-flow ischaemia, before reperfusing for 6 min and the succinate measured in the perfusate collected during each minute of reperfusion. Control reperfusion from *Figure 2B* (*n* = 5) and non-specific transport inhibitors added at 1 mM succimer (*n* = 3) and 1 mM phenylsuccinate (*n* = 3) at the onset and during reperfusion. (*C*) Mouse hearts were exposed to ischaemia as in *Figure 5B* but reperfused with buffer at pH 6 (*n* = 5). (*D*) Diagram of the mechanism of action of ionophores gramicidin, monensin, and nigericin. (*E*) Mouse hearts were exposed to ischaemia as in *Figure 5B* but reperfused in the presence of ionophores for 6 min (all at 10 µM): gramicidin (*n* = 5), monensin (*n* = 4), and nigericin (*n* = 4). All data (*B*, *C*, and *E*) are presented as mean ± SEM and statistical significance was assessed by two-way ANOVA with Dunnett’s *post hoc* test (*P <* *0.05, **P <* *0.01, ***P <* *0.001, ****P <* *0.0001 relative to control reperfusion).

The charged nature of succinate at physiological pH (pK_a_ 4.2 and 5.6) and the selectivity of its efflux upon reperfusion of ischaemic tissue suggests that its release is carrier-mediated. To assess this hypothesis, we measured the effect on succinate efflux of the general succinate transport inhibitors succimer and 2-phenylsuccinate, which are structurally similar to succinate (*Figure 5A*).^24^^,^^25^ Both these inhibitors decreased succinate efflux (*Figure 5B*), with a concomitant increase in succinate retention within the heart at the end of the reperfusion period (Supplementary material online, *Figure S2*).

### 3.5 Succinate efflux is enhanced by the plasma membrane proton gradient

The pH of the ischaemic myocardium can decrease to ∼6.5, compared to a pH of 7.4 for the heart perfusate.^26^^,^^27^ Hence, during reperfusion of the ischaemic heart there will be a pH gradient of ∼1 pH unit across the plasma membrane, acidic inside. As many metabolite transport processes are coupled to proton movement, we next assessed whether this pH gradient affects succinate efflux.^28^^,^^29^ To do this, we reperfused the ischaemic heart with pH 6 perfusion buffer to abolish the pH gradient (*Figure 5C*). Strikingly, this intervention greatly decreased succinate efflux (*Figure 5C*). To further assess the role of the pH gradient in succinate efflux we used a range of ionophores—gramicidin (H^+^, Na^+^, K^+^), monensin (H^+^, Na^+^), and nigericin (H^+^, K^+^), all of which can disrupt the plasma membrane pH gradient (*Figure 5D*). As these ionophores are all large hydrophobic molecules they act by inserting into the plasma membrane and are unlikely to redistribute to intracellular membranes such as the mitochondrial inner membrane over the timescale of these experiments.^30^ Thus, their effects are primarily due to disruption to the plasma membrane pH gradient. All these ionophores significantly decreased succinate efflux (*Figure 5E*). These ionophores are large hydrophobic molecules which are unlikely to migrate from the plasma membrane to disrupt mitochondrial membranes.

In contrast, use of the small protonophores carbonyl cyanide-4-(trifluoromethoxy)phenylhydrazone (FCCP) and 2,4 dinitrophenol (DNP) significantly disrupted cardiac function (data not shown). We conclude that succinate efflux upon reperfusion of the ischaemic heart is greatly enhanced by the pH gradient.

### 3.6 Succinate efflux from the reperfused ischaemic heart is mediated by MCT1

The above data show that succinate efflux upon reperfusion of the ischaemic heart is carrier-mediated and enhanced by a pH gradient. MCT1, which usually transports lactate in symport with a proton, is a potential carrier for this process.^28^^,^^31^^,^^32^ As the first succinate pKa is ∼5.6,^33^ at the pH within ischaemic tissues (∼6.5)^26^^,^^27^ ∼10% of the succinate in the myocardium would be in the monocarboxylate form, which may be transported by MCT1.^6^^,^^28^^,^^34^ Supporting this possibility, expressing MCT1 in *Xenopus* oocytes led to succinate uptake into the cells, but only when incubated in a medium at acidic (pH ∼6) pH.^28^ Furthermore, MCT1 is highly expressed in heart tissue.^16^

**Figure 6:**
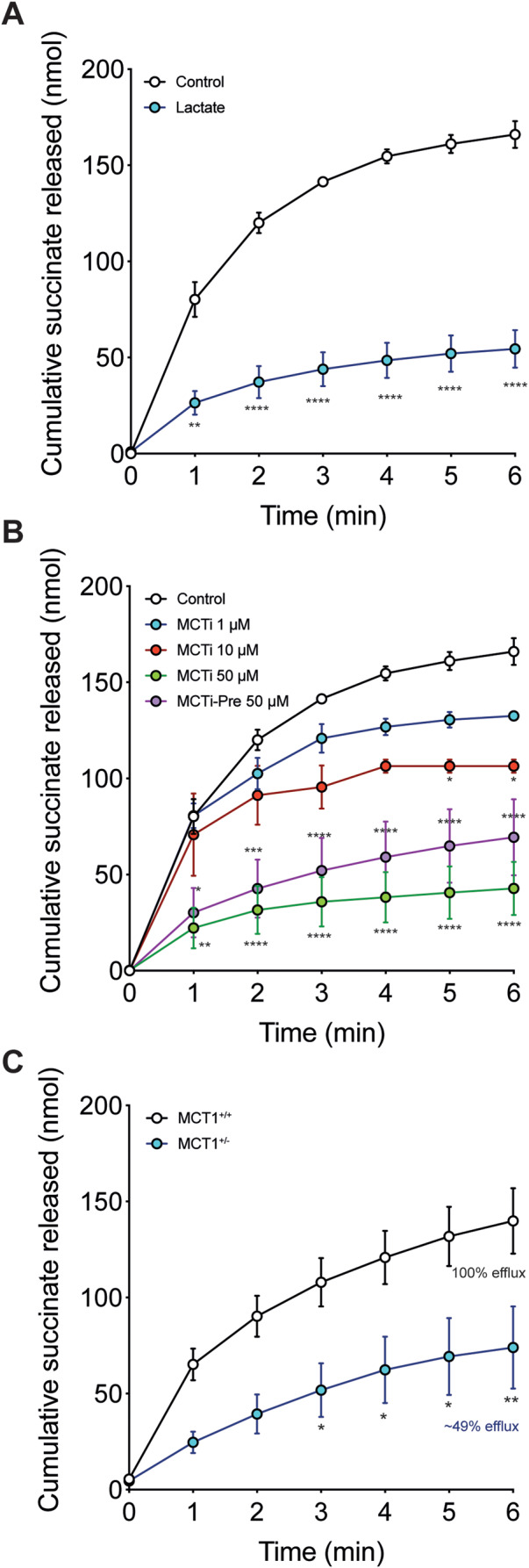
Succinate efflux during reperfusion is mediated by MCT1. (*A* and *B*) Langendorff hearts were treated as in *Figure 5* but reperfused in the presence of either (*A*) 10 mM L-lactate (*n* = 5) or (*B*) 1, 10, or 50 µM AR-C141990 (MCTi; *n* = 3–5). AR-C141990 was also used as a pre-treatment by infusing it during the equilibration phase prior to inducing ischaemia (MCTi-Pre at 50 µM; *n* = 5) (all mean ± SEM). Statistical significance was assessed by two-way ANOVA with Dunnett’s *post hoc* test (*P <* *0.05, **P <* *0.01, ***P <* *0.001, ****P <* *0.0001 relative to standard reperfusion). (*C*) Succinate efflux from *MCT1*^+/−^ and *MCT^+/+^* mouse hearts exposed to 20 min ischaemia upon reperfusion (mean ± SEM, MCT^+/+^*n* = 7, MCT^+/−^*n* = 5). Statistical significance was assessed by two-way ANOVA with Dunnett’s *post hoc* test (*P <* *0.05, **P <* *0.01 relative to *MCT1^+/+^* reperfusion). MCTi, monocarboxylate transporter 1 inhibitor.

To assess whether MCT1 could mediate succinate efflux in the reperfused ischaemic heart, we included lactate in the perfusion buffer to inhibit the activity of MCT1. This decreased succinate efflux (*Figure 6A*). Next, we reperfused ischaemic hearts with AR-C141990, a selective MCT1 inhibitor (reffered to as MCTi in the text),^35^^,^^36^ which led to a dose-dependent decrease in succinate efflux when added to the reperfusion buffer (*Figure 6B*). A similar effect was seen when hearts were administered the MCTi before ischaemia (*Figure 6B*), which did not alter the ischaemic levels of succinate (Supplementary material online, *Figure S3*). The MCTi also enhanced succinate retention within the reperfused tissue (Supplementary material online, *Figure S4*). Succinate efflux was also reduced by MCT1 inhibition with the MCTi, when it was added upon reoxygenation of anoxic cardiomyocytes. Here, it decreased succinate efflux by ∼90% from 1.28 ± 0.25 to 0.14 ± 0.02 nmol succinate/10^5^ cells (mean ± SEM, *n* = 6).

To confirm that MCT1 mediates succinate efflux from the ischaemic heart upon reperfusion, we utilized the *MCT1*^+/−^ mouse model.^16^^,^^37^^,^^38^ While the *MCT1*^−^^*/*^^−^ mouse is embryonic lethal, *MCT1*^+/−^ mice have ∼40% reduction in the expression of MCT1 in the heart^16^ and have no obvious phenotype in normoxic conditions when compared to *MCT^+/+^* controls.^16^^,^^37^^,^^38^ While the levels of succinate in the *MCT1*^+/−^ and *MCT^+/+^* hearts were the same after 20 min ischaemia (Supplementary material online, *Figure S3*), succinate efflux from reperfused *MCT1*^+/−^ hearts was significantly lower than that of *MCT1^+/+^* control hearts (*Figure 6C*). The levels of succinate retained in the heart after 6 min of reperfusion did not differ between the *MCT1^+/+^* and *MCT1*^+/−^ hearts (Supplementary material online, *Figure S5*). This result suggests that the reduction in MCT1 level in the *MCT1*^+/−^ hearts is sufficient to decrease succinate efflux.

## 4. Discussion

**Figure 7:**
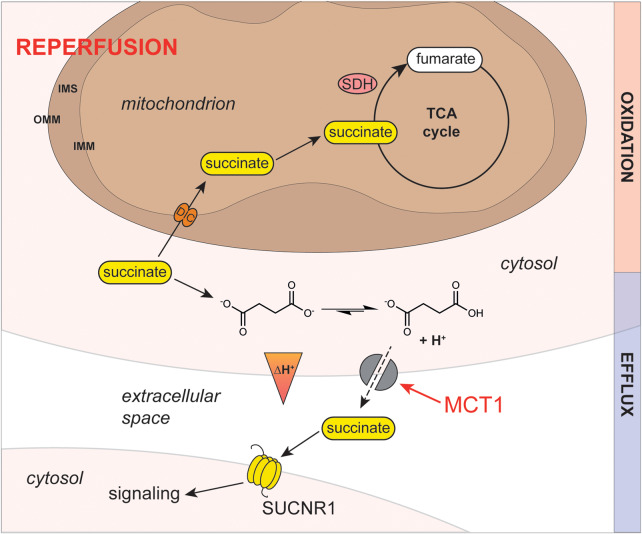
Model of the mechanism of succinate efflux from the ischaemic heart during reperfusion. In the ischaemic cardiomyocyte, succinate protonation to a monocarboxylate is favoured by the more acidic intracellular pH. During reperfusion, the intracellular pH is restored by proton efflux due to the proton gradient across the plasma membrane. Here, succinate monocarboxylate together with a proton is effluxed from the cardiomyocyte by MCT1, reducing intracellular succinate levels. Succinate release upon reperfusion may be a signal of tissue ischaemia and/or damage. SUCNR1 is a G-protein-coupled succinate receptor that can respond to the succinate released into the circulation. SUCNR1 is highly expressed on the surface of immune cells and its ligation has been associated with a range of both pro- and anti-inflammatory phenotypes, depending on context. IMM, inner mitochondrial membrane; IMS, inter-membrane space; MCT1, monocarboxylate transporter 1; OMM, outer mitochondrial membrane; SDH, succinate dehydrogenase; SUCNR1, G-protein-coupled succinate receptor; TCA, tricarboxylic acid.

The accumulation of succinate during ischaemia and its oxidation upon reperfusion is a key driver of I/R injury.^1–5^ While succinate is made within the mitochondrial matrix during ischaemia, most succinate will be present in the cytosol at the onset of reperfusion. In addition, it is also clear that upon reperfusion some of the succinate accumulated during ischaemia is released from the tissue upon reperfusion.^2^^,^^6^ Here, we show that this succinate efflux from the cell is greatly enhanced by the pH gradient (acidic inside) between the tissue and the circulation upon reperfusion, and that this efflux is mediated by the monocarboxylate transporter, MCT1. The best-understood role of MCT1 is as a plasma membrane lactate transporter, which leads to the electroneutral efflux of lactate along with a proton. At the low pH (∼6.5) within ischaemic tissue,^26^^,^^27^ ∼10% of the succinate will be in the monocarboxylate form (pKa ∼ 5.6),^33^ which can then be transported by MCT1,^28^ presumably because of its similarity in structure to lactate. In addition, because MCT1 transports a monocarboxylate in symport with a proton, the pH gradient between the tissue and the circulation present upon reperfusion of the ischaemic tissue will also drive succinate efflux. This model of succinate efflux, which was first suggested by Halestrap and colleagues^28^ is shown in *Figure 7*. Importantly, this work shows that the succinate accumulated in the heart during ischaemia has two fates: it is either oxidized by the mitochondrial respiratory chain or it is released into the circulation, potentially acting as a metabolic signal. A precedent for such signalling is when circulating succinate is taken up and activates thermogenesis in brown adipose tissue.^14^ Thus, elucidating the mechanism of succinate efflux from the ischaemic heart during reperfusion raises the prospect of targeting MCT1-dependent succinate in heart attack. The kinetics and current understanding of succinate transport by MCT1 is poor. While some initial characterization of this process has been carried out,^28^ more complete experiments are required to understand the interplay of the pH gradient and succinate transport.

There are a number of experimental points that should be considered in interpreting our findings. Perfused heart experiments have a number of limitations, such as the use of a supra-physiological concentration of glucose and the lack of fatty acids or other physiological respiratory substrates.

However, it should be noted that an isolated perfused heart which was supplied with fatty acids as an energy source accumulates succinate to a similar extent as hearts perfused with glucose-containing medium.^4^ The second is that ischaemic hearts *in vivo*, which had been perfused with blood prior to ischaemia and were thus respiring on physiological substrates, also accumulate succinate to the same extent as the isolated perfused heart.^4^^,^^5^ The third point is that reperfusion of the ischaemic human heart,^2^ or pig heart (this work), *in vivo* with blood leads to similar efflux of succinate. Thus, our *in vitro* heart perfusion system shows similar succinate accumulation during ischaemia and efflux upon reperfusion as the heart *in vivo*.

The potential role of succinate efflux as a signal from the ischaemic tissue is supported by the fact that its efflux is carrier-mediated, that succinate accumulates dramatically within ischaemic tissues, and that the lowered pH within ischaemic tissues facilitates succinate efflux. The accumulation of succinate during ischaemia seems to be a universal phenomenon and has now been shown by us and many others for hearts from mice, rats, rabbits, pigs, and humans.^1–5^ Importantly, this accumulation of succinate during ischaemia also occurs *in vivo* within tissues utilizing endogenous substrates, as well as in the Langendorff model, presumably because during ischaemia the heart relies on glycogen as its main energy source.^1–5^^,^^39^

The succinate efflux from the ischaemic mouse hearts *in vivo* and *ex vivo* was associated with a limited number of other metabolites. The metabolome of the pig heart attack model mirrored that seen in STEMI patients,^2^ suggesting a conserved mechanism of release of these metabolites from the ischaemic heart during reperfusion, with succinate being particularly elevated in both pigs and humans. We note that the pig is a widely used model for human cardiac metabolism, and importantly these were young, healthy animals analysed under tightly controlled conditions, compared to the human subjects^1^^,^^2^ who were all suffering from cardiac disease. Hence these data indicate that the succinate efflux in the human subjects was not simply a consequence of pathology. Lactate was prominent in the perfusate from mouse, a metabolite surprisingly not significantly elevated in the human plasma.^2^ This difference may be due to global ischaemia in the Langendorff heart compared to regional ischaemia in the heart attack models, or due to the high levels of lactate already present in the plasma masking changes.

The action of succinate ligating its cognate receptor, SUCNR1, and subsequent immune activation during I/R injury by succinate released upon reperfusion may contribute to the damage associated with I/R injury (*Figure 7*).^8^^,^^9^^,^^40^^,^^41^ However, the pathological role of succinate efflux and the signalling that occurs on ligation to SUCNR1 are currently unknown.

Furthermore, inhibition of succinate efflux upon reperfusion with MCTis might be expected to elevate tissue succinate oxidation and thus exacerbate I/R injury. To test this possibility, we carried out a preliminary experiment to assess the effect of the MCTi AR-C141990 on cardiac I/R injury in an *in vivo* mouse model of cardiac I/R injury (Supplementary material online, *Figure S6*). Administration of the MCTi upon reperfusion was protective, despite the elevated tissue levels of succinate it caused in the isolated perfused heart (Supplementary material online, *Figure S4)*. Further work is required to understand the mechanistic basis of this protection. One possibility is that the lack of lactate efflux has an impact on the cell, perhaps by its impact on glycolytic flux or cell pH. One possibility is that these factors disrupt mitochondrial succinate oxidation or ROS production during reperfusion. Future work will be required to determine fully the (patho)physiological roles of MCT1-dependent succinate efflux.

## Data availability

The data underlying this article will be shared on reasonable request to the corresponding author.

## Supplementary material

Supplementary material is available at *Cardiovascular Research* online.

## Authors’ contributions

H.A.P, M.P.M., and D.A. conceived and designed the studies. D.A. carried out Langendorff perfusions. M.M.H and T.E.B. carried out *in situ* I/R perfusions with guidance from K.S.-P. J.F.M. carried out *in vivo* LAD model supervised by T.K. A.V.G. carried out cardiomyocyte experiments. H.A.P. carried out succinate quantification and ROS measurements. T.Y., L.T., and E.N. carried out metabolomics with guidance from C.F. R.A. carried out pig I/R experiments. A.H. and L.P. developed MCT1^+/−^ mouse model and genotyping. M.J.S. provided Langendorff heart perfusion equipment and facilities for MCT^+/-^ experiments. All authors interpreted data. H.A.P., M.P.M., and D.A. wrote the original manuscript and all authors reviewed the revised manuscript.

## Supplementary Material

cvaa148_Supplementary_Data
