# Supplementary material for: Mechanism of succinate efflux upon reperfusion of the ischaemic heart
Source: Cardiovasc Res. 2020 Aug 7;117(4):1188–201. doi: 10.1093/cvr/cvaa148 (PMC7983001; doi:10.1093/cvr/cvaa148)

**ORIGINAL ARTICLE**

**Mechanism of succinate efflux upon reperfusion of the ischemic heart**

**Hiran A. Prag^1,2†^, Anja V. Gruszczyk^1,3†^**, **Margaret M. Huang^3^**, **Timothy E. Beach^3^**, **Timothy Young^2,4^**, **Laura Tronci^4^**, **Efterpi Nikitopoulou^4^**, **John F. Mulvey^2^, Raimondo Ascione^5^**, **Anna Hadjihambi^6^**, **Michael J. Shattock^7^**, **Luc Pellerin^6,8,9^**, **Kourosh Saeb-Parsy^3^**, **Christian Frezza^4^**, **Andrew M. James^1^**, **Thomas Krieg^2^**, **Michael P. Murphy^1, 2^**, **and Dunja Aksentijević^7,10^***

^1^ MRC Mitochondrial Biology Unit, Biomedical Campus, University of Cambridge, Cambridge CB2 0XY, UK

^2^ Department of Medicine, University of Cambridge, Cambridge, CB2 0QQ, UK

^3^ Department of Surgery and Cambridge NIHR Biomedical Research Centre, Biomedical Campus, University of Cambridge, Cambridge, CB2 2QQ, UK

^4^ MRC Cancer Unit, University of Cambridge, Hutchison/MRC Research Centre, Box 197, Cambridge Biomedical Campus, Cambridge, CB2 0XZ, UK

^5^ Bristol Medical School and Translational Biomedical Research Centre, Faculty of Health Science, University of Bristol, Level 7, Bristol Royal Infirmary, Upper Maudlin Street, Bristol, BS2 8HW, UK

^6^ Département de Physiologie, Université de Lausanne, 7 Rue du Bugnon, 1005 Lausanne, Switzerland

^7^King’s College London, British Heart Foundation Centre of Excellence, The Rayne Institute, St Thomas’ Hospital, London SE1 7EH, UK

^8^Centre de Résonance Magnétique des Systèmes Biologiques, UMR5536 CNRS, LabEx TRAIL-IBIO, Université de Bordeaux, Bordeaux Cedex, 33760, France

^9^Inserm U1082, Université de Poitiers, Poitiers Cedex, 86021, France

^10^ School of Biological and Chemical Sciences, Queen Mary University of London, G.E. Fogg Building, Mile End Road, London, E1 4NS, UK

*Corresponding author. Tel: +44 (0)20 7882 6973; E-mail: d.aksentijevic@qmul.ac.uk

**^†^** These authors contributed equally to the study

**Word count:** 8768 (including abstract, main text, figure legends & references)

**Running title:** Succinate efflux from the reperfused heart

**SUPPLEMENTARY FIGURE LEGENDS**

**Figure S1** Succinate efflux in a porcine MI model only occurs from the ischemic tissue. Pigs were treated as in *Figure 4E*: **s**uccinate is elevated during early reperfusion in the coronary sinus plasma but not jugular vein or aortic root in a pig heart attack model. The LAD was occluded by a balloon catheter for 60 min before removing the occlusion and blood sampled (mean ± S.E.M., n=3). Statistical significance was assessed by two-way ANOVA with Tukey’s post hoc test (***p* <0.01, *****p* <0.0001).

**Figure S2** Succinate is retained in the heart when reperfused with nonspecific transport inhibitors. Succinate levels in hearts after 6 min reperfusion with 1 mM succimer or 1 mM phenylsuccinate (Phsucc) from *Figure 5B* were measured (mean ± S.E.M., n=3-5). Statistical significance was assessed by two-way ANOVA with Dunnett’s post hoc test (*****p* <0.0001 relative to control reperfusion).

**Figure S3** MCT1 inhibition or haploinsufficiency does not affect succinate accumulation.

Hearts were perfused in Langendorff mode and equilibrated with Krebs buffer alone, or for MCTi-pre with 50 µM AR-C141990 for 20 min, before 20 min global no-flow ischemia and snap freezing tissue for succinate quantification by LC-MS/MS (mean ± S.E.M or mean ± range for *MCT1^+/-^*; WT n=8 (from *Figure 2A*), *MCT1^+/-^* n=2, MCTi-pre n=4).

**Figure S4** Succinate is retained in the heart when reperfused with MCTi. Succinate levels in hearts after 6 min reperfusion with MCTi from *Figure 6B* were measured (mean ± S.E.M., n=3-5). Statistical significance was assessed by two-way ANOVA with Dunnett’s post hoc test (*****p* <0.0001 relative to control (ctl) reperfusion).

**Figure S5** Succinate retained in the heart after reperfusion in *MCT1^+/-^* mice is no different from *MCT1^+/+^* hearts. Succinate levels in *MCT1^+/+^* and *MCT1^+/-^* hearts after 6 min reperfusion from *Figure 6C* were measured (mean ± S.E.M., *MCT1^+/+^* n=7, *MCT^+/-^* n=5).

**Figure S6** Inhibition of MCT1 with AR-C141990 decreases cardiac ischemia-reperfusion injury. Anesthetized mice were subjected to occlusion of the LAD for 30 min and then the occlusion was removed and the hearts were reperfused for 120 min. The mice were infused IV for 20 min from 5 min before reperfusion with either saline, or saline supplemented with the MCT1i AR-C141990, at a total delivered dose of 1.5 mg/kg body weight. Infarct sizes were determined histologically as a percentage of risk area. Data are mean ± S.E.M., n=5. Statistical significance was assessed by unpaired, two-tailed Student’s t-test where **p*<0.05.

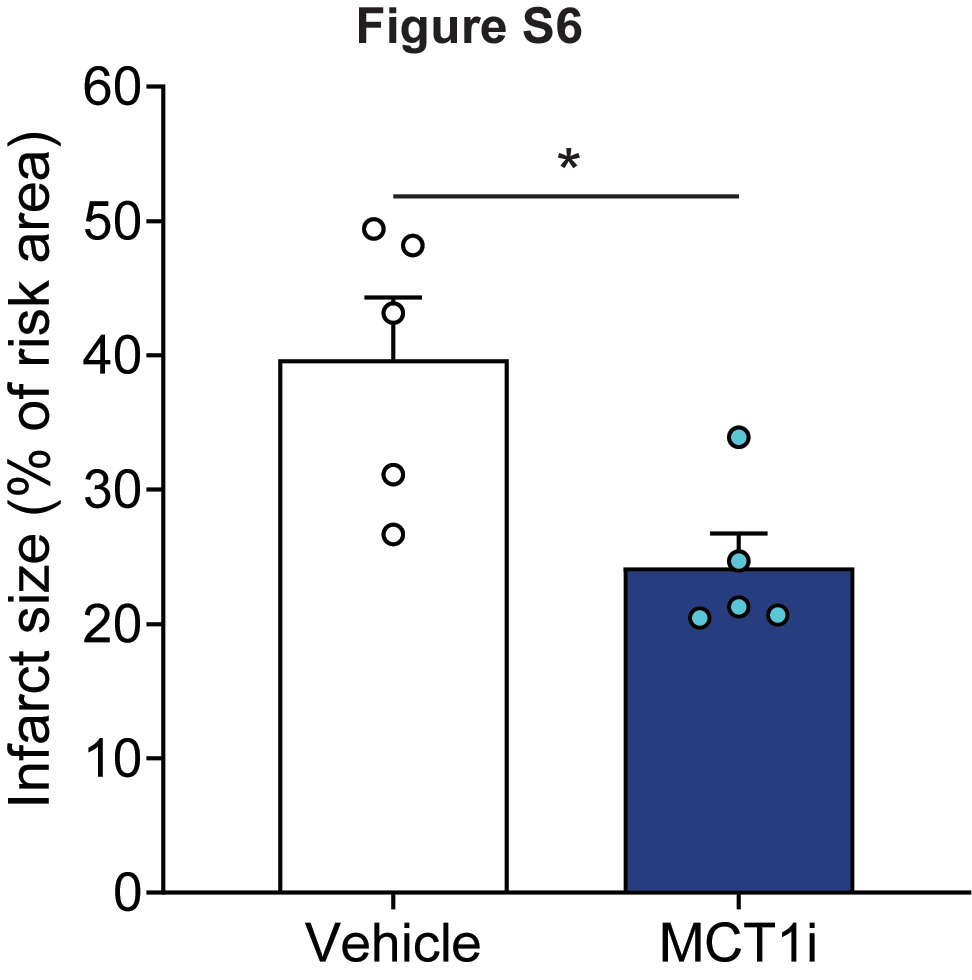

Supplement: cvaa148_Supplementary_Data [file cvaa148_supplementary_data.docx]
